# Personal, environmental and behavioral predictors associated with online fraud victimization among adults

**DOI:** 10.1371/journal.pone.0317232

**Published:** 2025-01-29

**Authors:** Vimala Balakrishnan, Umayma Ahhmed, Faris Basheer

**Affiliations:** Faculty of Computer Science and Information Technology, Universiti Malaya, Kuala Lumpur, Malaysia; Lucian Blaga University of Sibiu: Universitatea Lucian Blaga din Sibiu, ROMANIA

## Abstract

**Background:**

Online malicious attempts such as scamming continue to proliferate across the globe, aided by the ubiquitous nature of technology that makes it increasingly easy to dupe individuals. This study aimed to identify the predictors for online fraud victimization focusing on Personal, Environment and Behavior (PEB).

**Methods:**

Social Cognitive Theory (SCT) was used as a guide in developing the PEB framework. Specifically, three factors were identified—Self-awareness (Personal), Attitude (Personal and Environment) and Safe Practice (Behavior) as the potential predictors for online fraud victimization. A self-reporting questionnaire was developed based on the PEB framework and used to collect data targeting Malaysian adults. The study reports result from two separate datasets collected across two separate timelines. Study I involved data collection in January 2023 (n = 820) whereas Study II was conducted with a modified questionnaire from November 2023 –January 2024 (n = 629). Study I identified the online fraud victimization predictors through an Exploratory Factor Analysis (EFA) and a hierarchical binary logistic regression. The dataset from Study II was used to validate the online fraud victimization model derived from Study I by executing another round of hierarchical binary logistic regression.

**Results:**

Results from both the samples show that most of the respondents are aware of digital privacy. EFA from Study I yielded a five-factor solution with a total variance of 60.6%, namely, Self-awareness, Safe Practice, Bank Trust, Overconfidence and Social Influence. Hierarchical binary logistic regression results from both the studies were found to be consistent. Specifically, Overconfidence (β = 0.374; OR = 1.453; 95% CI [1.119, 1.887]; p = 0.005) and Social Influence (β = 0.332; OR = 1.225; 95% CI [1.077, 1.512]; p = 0.006) were found to significantly predict online fraud victimization as well as gender (β = 0.364; OR = 1.440; 95% CI [1.008, 2.016]; p = 0.045) with females exhibiting higher risks to victimization.

**Implications:**

The emergence of Overconfidence and Social Influence as significant predictors can guide the development of targeted online fraud awareness campaigns and/or tools emphasizing critical thinking and skepticism. Policymakers can leverage this knowledge to implement regulations that reduce deceptive practices online, promote digital literacy programs, and mandate clearer consumer protections to mitigate the impact of social manipulation and overconfidence on fraud victimization.

**Conclusion:**

This study identifies online fraud victimization predictors, hence improving our understanding of the factors behind this phenomenon—allowing for the development of effective preventive measures and policies to safeguard individuals and improve digital security. For instance, gender- specific educational campaigns can be developed to enhance awareness and equip women with strategies to detect and avoid scams. Additionally, addressing systemic factors like social norms and digital literacy gaps is crucial for creating equitable and effective solutions to reduce online fraud victimization.

## Introduction

The Internet and digital technology have become an integral part of our daily lives, a trend that is further amplified by the COVID-19 pandemic [1, 2]. Online consumer behavior witnessed a rapid change with majority of people resorting to digital technologies for most routine transactions including shopping, bill payments and procuring services, among others. This also includes the accelerated use of online platforms such as social media, as avenues to seek information, provide support, and to build relationships. However, these may have inadvertently increased the digital attack surface and amplified issues related to digital security and privacy. An increase in fraudulent activities ranging from scamming, phishing to data breach was observed worldwide. These activities are often accomplished through “social engineering” [3]–influencing/tricking people to perform unsafe actions such as clicking on fake website links, divulging personal and confidential details (e.g., username and passwords), opening malware enabled attachments, and downloading untrusted third-party apps for fraudulent payment transactions etc. for malicious gains [4].

Malaysia too, has witnessed a healthy rate of digitization following the COVID-19 outbreak. Reports on online fraud activities, particularly scamming are rampant worldwide, a trend that is considered worrying in Malaysia. For instance, there were 13,703 reported scam cases in the country involving losses of MYR 539 million (USD 124 million), a figure that increased to 20,701 reported cases with losses amounting to MYR 560.8 million (USD 129 million) in 2021 [5]. Most of these cases were associated with online trading, fake loans, Short Message Service (SMS) scams, African/Nigerian (request to help facilitate the illegal transfer of money) and Macau (tricking someone into disclosing personal banking details or transferring money into third party accounts) scams. The increase in such fraudulent activities was attributed to rising expertise in technology and low level of awareness on cybercrimes among the people [6].

The rise in digital technologies that are most often integrated, means online fraudulent activities to be a growing issue across any society. However, the present understanding of what makes an individual susceptible to online fraud is relatively poor. The concept of digital privacy is broad regardless of the platform. In the context of social media, for example, privacy may refer to one’s tendency to freely disclose information (public versus private), or concern about how personal data is being used (or misused) by others. It is pertinent to understand why online fraudulent victimization is on the rise, and this is probably best done by exploring some basic themes, namely, awareness, attitude and behavior towards digital privacy. The study therefore aims to explore predictors leading to online fraud victimization by focusing on personal, environment and behavior–the three main tenets of Social Cognitive Theory (SCT). Specifically, we aim to answer two main research questions (RQs):

RQ1: What is the awareness level of digital privacy among Malaysian adults?

RQ2: What are the significant predictors for online fraud victimization?

### Theoretical framework and research hypotheses

This empirical study is guided by a theoretical framework encompassing three main factors, namely, Self-awareness, Attitude and Behavior, developed based on SCT that emphasizes on people, who are seen as active agents who can both influence and are influenced by their environment [7]. Specifically, SCT proposes that human behavior is influenced by three key tenets: personal such as individual beliefs, attitude, values etc., environment such as social norms and cultural influences, and behavioral including skills and abilities. For example, Self-awareness is deemed to be a critical personal factor that can influence decision making by affecting how individuals perceive and interpret information. According to SCT, this enables individuals to make informed decisions as they are more aware of their actions and potential consequences of their decisions.

According to Fenigstein et al. [8], the disposition for self-focused attention is an individual difference factor related to resistance to any form of influence. Individuals with a high sense of self-awareness, for example, are known to consider their personal knowledge, internal norms and attitudes to a greater degree when making decisions and thus leading to increased resistance to social influence and persuasion attempts [9]. Studies have shown that being aware of the risks associated with online mechanisms as well as the tactics used by fraudsters can help individuals be more vigilant, and help identify and assess risks, hence they are better equipped in protecting themselves [10–13]. Therefore, we posit that individuals with high self-awareness are less susceptible to online fraud victimization. Hypothesis one is given as follows:

H1 –Self-awareness negatively predicts online fraud victimization

Another personal factor that is known to affect individuals’ behavior and decision-making is attitude, which refers to individual characteristics, portraying either positive, neutral or negative behavior to a certain concept or subject [14]. It can be influenced by several factors such as social norms (i.e., environment) personal values, beliefs and experiences. Attitude has been well-studied in various domains, ranging from those focusing on threat appraisal and coping strategies of online fraud [12, 13], technology adoption [15–17], to social issues including misinformation spread [18] and online hate [19]. These studies generally show that a positive attitude promotes good behavior, and vice-versa.

In the context of digital privacy, a negative attitude towards sharing personal information online was found to significantly predict phishing susceptibility [12, 13]. Similar findings were reported by those focusing on self-efficacy and online scams [20, 21]. We hypothesize that a negative attitude such as being overconfident or lackadaisical can lead to online fraud victimization, as it may result in individuals being careless or causing them to make quick decisions without considering the potential consequences of their actions. Further, environment has a pertinent role in how an individual behaves or reacts to a situation. For example, some individuals have a higher propensity in trusting people, hence making them more susceptible to fraud victimization. As stated in Introduction, most online fraud activities are done through social engineering [3, 22, 23] by gaining the trust of victims, and thus individuals who are overly trusting are more vulnerable to manipulation and may less likely take precautions to protect themselves from fraud/fraudsters. In this study, attitude covers a wide range of elements including personal and environment factors. In general, hypothesis two is given as below:

H2 –A negative Attitude positively predicts online fraud victimization

Finally, the third factor is user behavior. Though this may encompass a wide spectrum of behavior, this study is specifically interested in secure or safe online practices adopted by individuals as a means of protection. For example, making it a norm to set and use strong passwords containing numbers, upper- and lower-cases, and/or special symbols, ensuring computer and mobile phone software are up to-date, being vigilant when using the public wi-fi or using a virtual private network (VPN) are some of the safe measures one can adopt to ensure internet security. As a matter of fact, studies have shown that adopting and practicing safe measures reduces breach of security, online scamming and other fraudulent activities [24–27]. Hypothesis three therefore, posits that individuals who practice safe measures are less susceptible to online fraud.

H3 –Safe Practice negatively predicts online fraud victimization

## Materials and methods

**Fig 1:**
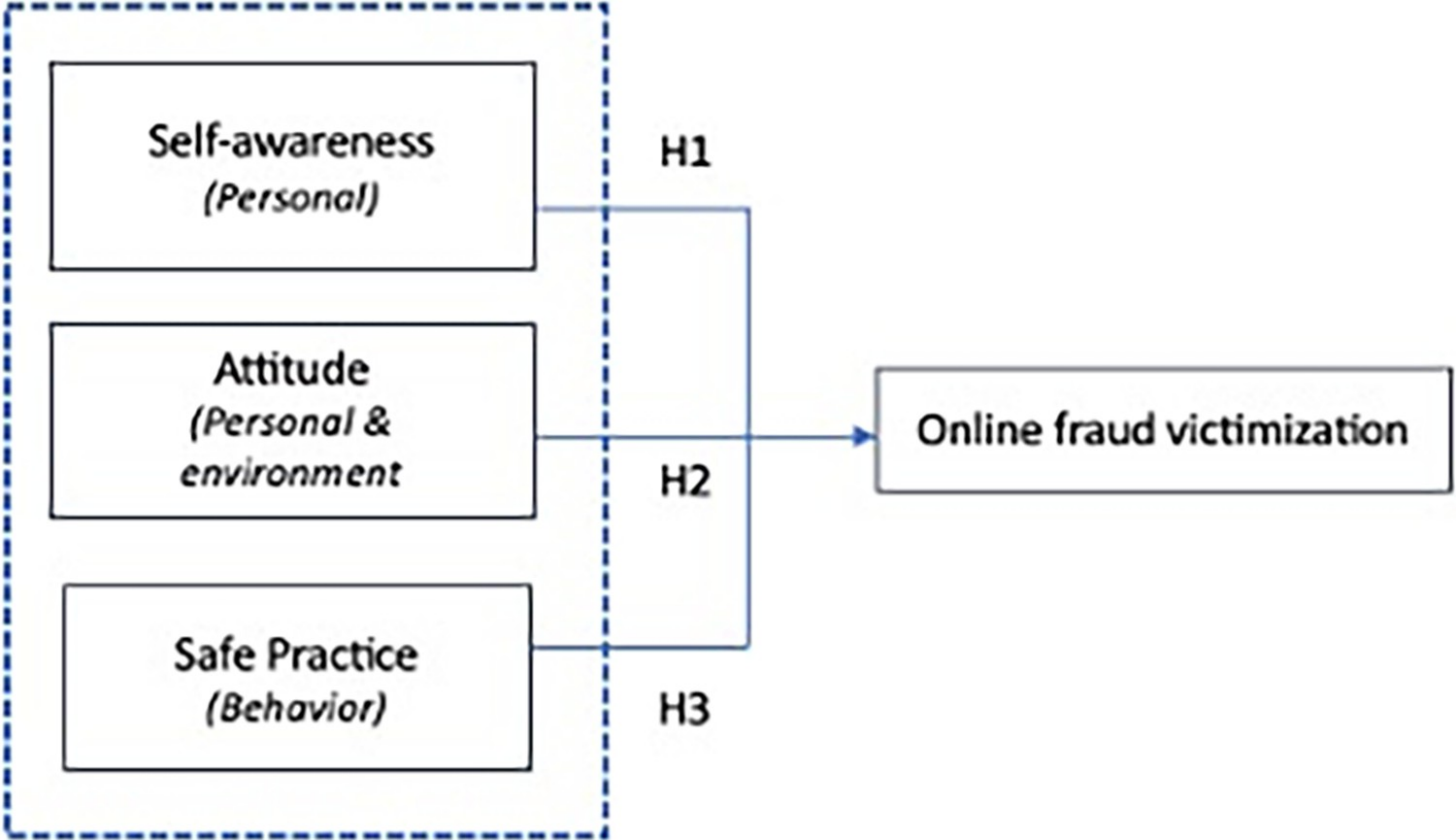
Conceptual framework for online fraud victimization.

This study explored the predictors for online fraud victimization by focusing on personal, environment and behavioral factors based on SCT. The conceptual framework was built based on previous literatures, specifically focusing on Self-awareness [10, 12, 13, 28], Attitude [12, 13, 20, 21] and Safe Practice [11, 25, 26], as illustrated in **Fig 1.**

Data collection, analysis and validation were done over the course of two timelines (i.e., January 2023 –January 2024), resulting in two sets of data. We differentiate the studies as Study I and II.

## Study I

### Study design and sample

The initial study (Study I) aimed to identify the predictors for online fraud victimization. The data collection was conducted from January 31 to February 23, 2023, using a self-administered online survey hosted on the Google Forms platform. The link to the survey was distributed through social media, and WhatsApp. Using a calculator based on Cochran’s equation [29], the appropriate sample size given the specified combination of precision (±5%), confidence (95%) and variability (0.5) was 385. Targeting Malaysian adults (i.e., 18 and above), a total of 820 respondents were recruited.

### Instrument

A questionnaire was prepared in English and piloted among 36 respondents. Ambiguities were resolved, questions were rephrased and restructured to enhance the readability of the survey instrument. This was then translated into the national language (i.e., Malay), and verified by four linguistic experts. Both versions were provided to the respondents, with an option to pick one. The instrument had two main sections:

Section A–This was used to gather respondents’ demographic details such as age (continuous), gender, ethnicity, hours spent daily on social media etc. Respondents were also asked to rate their digital literacy (beginner, intermediate, advanced) and online risky behavior levels (low, medium, high). The level of digital privacy awareness was assessed through a single question (i.e., are you aware of digital privacy?). Further, another question determined online fraud victimization (i.e., have you been a victim of any online fraud activity, e.g., scammed, data breach, identity theft etc.?), which was used as the dependent variable in this study. The complete items can be found in **Table 1 (Results).**

**Table 1:** Demographic profiles of the 820 respondents.

Characteristics	Sub-characteristics	Frequency	Percent
Age	Gen Z (18 -– 26)*	572	69.8
	GenY (27–42)	120	14.6
	Gen X (43–58)	117	14.3
	Baby Boomers (59–77)	11	1.3
Gender	Male	374	45.6
	Female	446	54.4
Status	Tertiary Students	535	65.2
	Working	266	32.4
	Others	19	2.3
Ethnicity	Malay	494	60.2
	Chinese	180	22
	Indian	121	14.8
	Others	25	3.0
Daily phone use (hours)	Less than 1	2	0.2
	Between 1–5	514	62.7
	More than 5	304	37.1
Daily social media (hours)	Less than 1	54	6.6
	Between 1–5	546	66.6
	More than 5	220	26.8
Aware of digital privacy?	Yes	669	81.6
	No	44	5.4
	Unsure	107	13.0
Have you been victimized?	Yes	344	42.0
	No	476	58.0
Victimization medium used	Social media	215	26.0
	3rd party app	52	6.3
	Chat applications	91	10.8
	Phone call/SMS	155	18.9
	Website	4	0.2
	Shopping apps	3	0.1
	Game	3	0.2
	Email	2	0.2
Attempt of being scammed?	No	153	18.7
	Yes	667	81.3
Technology literacy	Beginner	36	4.4
	Intermediate	460	56.1
	Advanced	324	39.5
Fake News literacy	Beginner	78	9.5
	Intermediate	516	62.9
	Advanced	226	27.6
Fact Check literacy	Beginner	194	23.7
	Intermediate	453	55.2
	Advanced	173	21.1
Online risky behavior level	Low	176	21.5
	Intermediate	515	62.8
	High	129	15.7

Note

*—Grouped according to the generation

Section B–This comprised three subsections measuring respondents’ perceptions, namely, attitude (n = 11; Cronbach’s alpha (α) = 0.87), behavior (n = 10; α = 0.76) and self-awareness (n = 11; α = 0.912). Example items for attitude were “I trust people easily”, “Online banking is safe”, “I will never fall victim as I am always careful” and “I trust the safety mechanisms adopted by my banks” etc. On the other hand, items for behavior focused on the safe/risky measures adopted such as “I always perform 2-step verification for my personal accounts”, “I browse online using a secure VPN”, and “I click on online links as they are generally safe” etc. whilst items for awareness include “I know the dangers of digital footprints” and “I am aware that my personal data are collected online”, among others. All the 32 items in Section B were measured using a five-point Likert scale indicating the extent of a respondent’s agreement with the items (5 = Strongly agree, 4 = Agree, 3 = Neutral, 2 = Disagree, and 1 = Strongly disagree). All the negative statements were re-coded prior to analysis. Cronbach’s alpha (α) was used to determine the internal consistency and reliability, and the scores for each of the factors ranged between 0.76 and 0.91 (i.e., > 0.7), hence indicating a good reliability. The complete questionnaire is attached as SI.

### Data analysis

Frequency, percentage, mean and standard deviations (SD) were used to describe the sample. Exploratory Factor Analysis (EFA) was used to examine the underlying factor structures whereas a hierarchical binary logistic regression was performed to identify the significant predictors for online fraud victimization. The analyses were conducted using IBM SPSS version 28. Results are deemed significant at p ≤ 0.05.

Skewness and kurtosis values indicate values between −1.98 and +1.98 [30, 31], hence the data were considered normally distributed. Preliminary analysis revealed no multicollinearity issues as the communalities were above 0.5, the inter-item correlations and item-to-total correlations were above the recommended values of 0.3 and 0.5, respectively [32]. Further, the Kaiser-Meyer-Olkin (KMO) of sampling adequacy score was 0.869, indicating sample size adequacy. The Bartlett’s Test of Sphericity also supported the factorability of the correlation matrix, with x2 = 9233.34; degree of freedom (df) = 465; p < 0.001 [33]. The number of factors were determined based on the Eigenvalues (i.e., more than 1.00) whilst items with a factor loading less than 0.5 were removed.

### Ethics approval

This study was approved by the Universiti Malaya Research Ethics Committee [UM.TNC 2/UMREC]. Written individual informed consent was obtained from all participants. All the data collection and analysis method used in this study complied with the terms and conditions for the source of the data.

## Results

### Demographics

Demographic profiles of the 820 Malaysian respondents are provided in **Table 1.** There was a total of 820 respondents aged between 18 and 67 years old (Mean = 27.14; SD = 11.4), mainly comprising Gen Z (69.8%; 18–26 years), followed by Gen Y (14.6%; 27–42 years) and Gen X (14.3%; 43–58 years), in line with the status whereby most of the respondents were tertiary students (65.2%). Gender segregation is balanced (male: 374 versus female: 446). Most of the respondents were also active as they spent between 1 and 5 hours on their phones (62.7%) and social media (66.6%) daily. Forty-two percent of the respondents reported to have been a victim of online fraud (at least once) (42%), of which the majority claimed the perpetration to have taken place on social media (26%) and phone calls/Short Messaging Services (SMS) (18.9%).

In terms of digital literacy, most of the respondents reported to be at the intermediate level for technology (56.1%), fake news literacy (62.9%) and fact-checking literacy (55.2%). A similar pattern was observed for online risky behavior level, with the majority reported to be intermediate (62.8%). Finally, Table 1 also shows that 81.6% (n = 669) are aware of digital privacy. This shows that the level of digital privacy awareness is high among our sample, hence answering our RQ1.

### Underlying factors for online fraud victimization

To answer RQ 2, an EFA was performed using all the items in Section B. This is then followed by a hierarchical binary logistic regression. Using Principal Component Analysis (PCA) with Varimax rotation, the EFA produced a five-factor solution, with a total variance of 60.6%. **Table 2** shows the results of EFA, along with mean, SD and α.

**Table 2:** Exploratory factor analysis results: Factor loadings.

Items	Self-awareness	Safe Practice	Overconfidence	Bank Trust	Social Influence
**Mean: 4.05; SD: 0.98:** α **= 0.912**					
I am aware that my online activities can be tracked without my knowledge	0.819				
I am aware that my online activities can be tracked without my permission	0.803				
I am aware that malicious websites may lead to identity theft (by installing a software on my PC without my notice to collect personal info)	0.779				
I am aware that my personal data are collected online	0.759				
I am aware that there are a lot of fake websites on the internet	0.752				
I know the dangers of sharing sensitive information online	0.736				
I know the dangers of digital footprints	0.694				
I know which suspicious pop-up messages on websites to be ignored	0.661				
I know that downloading suspicious app with APK is dangerous	0.654				
I know the importance of safety measures online	0.62				
I am aware of the after-effect of accepting cookies on websites	0.597				
**Mean: 3.41; SD: 1.05:** α **= 0.798**					
I always perform 2-step verification for my personal accounts		0.725			
I use auto save password settings on my phone		0.703			
I usually logout from mobile apps/social media/websites etc.		0.703			
I keep my antivirus software up to-date		0.624			
I make sure that my chat messages online are encrypted		0.613			
I use privacy settings to block malicious websites		0.607			
I always use a strong password		0.593			
**Mean: 3.12; SD: 1.04:** α **= 0.744**					
I am confident that my online data will remain private and confidential			0.758		
I believe I can fully control my privacy online			0.734		
I will never fall victim as I am always careful			0.661		
**Mean 3.01: SD 0.93:** α **= 0.801**					
I trust the safety mechanisms adopted by my banks				0.853	
Online banking is safe				0.849	
**Mean 2.46; SD 1.05;** α **= 0.65**					
I am easily manipulated by others					0.703
I tend to trust people easily					0.646
My family and friends influence the choice of apps that I use					0.621

All 11 items assessing awareness loaded into the first cluster, hence the factor’s name was retained as Self-awareness. The high mean score (4.05) indicates most of the respondents were agreeable in having a high sense of self-awareness towards digital privacy, a finding that accords with the level of digital privacy awareness reported in **Table 1.** Similarly, seven items focusing on behavior loaded into the second cluster, therefore the name was retained as Safe Practice. Three items were removed as they had a loading below 0.5, namely, “I change my passwords regularly”, “I use the same password for every account”, and “I browse online using a secure VPN”. The mean score of 3.41 generally indicates the respondents to be somewhat agreeable in adopting safe online measures.

Items measuring Attitude split three ways–three items focusing on an individual’s confidence (i.e., overconfidence) loaded together, therefore this was named as Overconfidence, two specifically focusing on trust in banks loaded together, hence this was named as Bank Trust. Finally, three items on impact of social influence and being overly trusting loaded together, and thus this was named as Social Influence. Three items were removed due to a low load, that is, “The Internet is safe”, “Scammers only target older generations”, and “I do not care about my privacy setting”. The α values for Overconfidence and Bank Trust were 0.74 and 0.80, respectively. The α value for Social Influence was 0.65, however, since a reliability score between 0.5 and 0.7 is deemed to be moderate, this factor was retained.

Based on the results of EFA, hypothesis two is rephrased as follows:

H2a –An overconfidence attitude positively predicts online fraud victimization
H2b –A high trust in banking institutions positively predicts online fraud victimization
H2c– A high social influence positively predicts online fraud victimization

#### Online fraud victimization predictors

Finally, a hierarchical binary logistic regression was performed to identify the significant predictors for online fraud victimization. The dependent variable was online fraud victimization (Yes = 0; No = 1). Frequency of social media use, being aware of digital privacy, gender (male = 0; female = 1) and online risky behaviour level were entered in Block 1 (Model 1), followed by all five factors from EFA in Block 2 (Model 2). We report on the results for Model 2 in **Table 3.**

**Table 3:** Study I–Hierarchical binary logistic regression for online fraud victimization.

Predictors	β	Wald	p	OR	95% CI for OR	
					Lower	Upper
Self-awareness	0.145	3.314	0.069	1.156	0.989	1.351
Safe Practice	0.097	1.618	0.203	1.101	0.949	1.278
Overconfidence	0.362	21.88	< .001*	1.437	1.234	1.673
Bank Trust	0.013	0.029	0.865	1.013	0.876	1.171
Social Influence	0.263	11.695	< .001*	1.169	1.061	1.594

Note

*significant at *p* < 0.05; All the demographic variables were insignificant, hence results are not shown in the table; OR: Odd-ratio

The overall model was found to be fit (x^2^ = 46.67; degree of freedom (df) = 16; p < 0.001), with a -2 log likelihood error of 1052.15 (Nagelkerke R^2^ = 7.6%; Hosmer and Lemeshow p-value = 0.807). The classification accuracy was 62% compared to 56.9% for Model 1, indicating an improvement in online fraud victimization prediction with the inclusion of the EFA factors.

Looking at the p-values in **Table 3**, two significant predictors were found for online fraud victimization, namely Overconfidence and Social Influence. A higher level of Overconfidence was associated with higher odds of online fraud victimization (β = 0.362; Odd-ratio (OR) = 1.437; 95% CI [1.234, 1.673]; p < 0.001), indicating that the more overconfident an individual is the more susceptible he/she is toward online fraud victimization. Similarly, results show that an individual to more likely fall victim online when there is a higher Social Influence (β = 0.263; OR = 1.169; 95% CI [1.061, 1.594]; p < 0.001). The rest of the predictors were insignificant. In short, H2b and H2c are supported.

## Study II

The second study was conducted to validate the findings from the previous study, particularly in determining the significant predictors for online fraud victimization. All the approaches outlined in Study I were replicated, with minor modifications to the questionnaire. Specifically, items that were removed during EFA in Study I were excluded (i.e., “I change my passwords regularly”, “I use the same password for every account”, “I browse online using a secure VPN”, “The Internet is safe”, “Scammers only target older generations”, and “I do not care about my privacy setting”. No other changes were made. Data collection took place from November 11 to January 20, 2024, resulting in 630 valid responses. However, one response was excluded for not meeting the study’s criteria (i.e., not being Malaysian), leaving a final sample of 629 respondents. The demographics of this group closely resembled those from Study I, as shown in **Table 4**, which provides a detailed demographic profile.

**Table 4:** Demographic profiles of Study II respondents (n = 629).

Characteristics	Sub-characteristics	n	%
Age	Gen Z (18 -– 26)	505	80.2
	GenY (27–42)	74	11.7
	Gen X (43–58)	38	6.04
	Baby Boomers (59–77)	12	1.9
Gender	Male	346	55
	Female	283	45
Status	Tertiary Students	522	83
	Working	93	14.8
	Others	14	2.2
Ethnicity	Malay	373	59.3
	Chinese	196	31.2
	Indian	58	9.2
	Others	2	0.3
Daily phone use (hours)	Less than 1	10	1.6
	Between 1–5	226	35.9
	More than 5	393	62.5
Daily social media (hours)	Less than 1	49	7.8
	Between 1–5	360	57.2
	More than 5	220	35
Aware of digital privacy?	Yes	523	83.1
	No	30	4.8
	Unsure	76	12.1
Have you been victimized?	Yes	201	32
	No	428	68
Victimization medium used	Social media	254	40.38
	3rd party app	89	14.15
	Chat applications	260	41.34
	Phone call/SMS	202	32.11
	Website	1	0.16
	Shopping apps	2	0.32
	Game	1	0.16
	Email	4	0.64
Attempt of being scammed?	No	94	14.9
	Yes	535	85.1
Technology literacy	Beginner	56	8.9
	Intermediate	401	63.8
	Advanced	172	27.3
Fake News literacy	Beginner	60	9.5
	Intermediate	385	61.2
	Advanced	184	29.3
Fact Check literacy	Beginner	136	21.62
	Intermediate	395	62.8
	Advanced	98	15.58
Online risky behavior level	Low	190	30.2
	Intermediate	364	57.9
	High	75	11.9

Generation segregation was found to be similar with most of the respondents from Gen Z (80.2%), followed by Gen Y (11.7%) and Gen X (6%). Gender segregation is balanced, however there were higher number of males (346) than females (283). Most of the respondents were also active on social media spending between 1 and 5 hours daily (57.2%). Thirty-two percent reported to have experienced online fraud victimization with social media, chat applications and phone calls/SMS emerging as the top three mediums. Digital, fake news and fact-checking literacy patterns as well as online risky behavior level were similar with most respondents reported to be at the intermediate level. As in Study I, many Study II respondents were aware of digital privacy (83.1%), followed by those who were unsure (12.1%) and those unaware (4.8%).

### Online fraud victimization prediction model

The hierarchical binary logistic regression also revealed Model 2 to be significant and fit (x^2^ = 22.96; df = 12; p = 0.028), with a -2 log likelihood error of 765.21 (Nagelkerke R^2^ = 5%; Hosmer and Lemeshow p-value = 0.743). A slight improvement was noted for the classification accuracy (68.2% compared to 67.6% for Model 1), resembling a similar pattern with Study I.

Model 2 produced similar results for the second dataset as shown in **Table 5**, with Overconfidence (β = 0.374; OR = 1.453; 95% CI [1.119, 1.887]; p = 0.005) and Social Influence (β = 0.332; OR = 1.225; 95% CI [1.077, 1.512]; p = 0.006) emerging as significant predictors. Additionally, gender was also found to be significant (β = 0.364; OR = 1.440; 95% CI [1.008, 2.016]; p = 0.045), indicating that females are more likely to be victimized online compared to males.

**Table 5:** Study II–Hierarchical binary logistic regression for online fraud victimization.

Predictors	β	Wald	p	OR	95% CI for OR	
					Lower	Upper
Gender (1)	0.364	4.021	0.045*	1.440	1.008	2.016
Self-awareness	-0.124	0.658	0.417	0.833	0.655	1.192
Safe Practice	-0.082	0.255	0.614	0.921	0.670	1.267
Overconfidence	0.374	7.851	0.005*	1.453	1.119	1.887
Bank Trust	0.061	0.286	0.593	1.063	0.849	1.332
Social Influence	0.332	7.563	0.006*	1.225	1.077	1.512

Note

*significant at p < 0.05; Only significant demographic variables are shown; Gender male = 0 (base); OR: Odd-ratio

## Discussion

Considering the spike in online fraud victimization cases, and the lack of understanding of the predictors leading to this phenomenon, this study explored the predictors associated using a self-administered questionnaire survey developed based on SCT. Using a sample size of 820 Malaysian adults, the proposed model was tested and further validated with a new dataset (n = 629) using hierarchical binary logistic regression. The study found Overconfidence, Social Influence and gender (female) to significantly predict online fraud victimization, whilst Bank Trust and Self-awareness did not. Further, a high level of digital privacy awareness was also observed among both the study respondents.

Findings indicate a positive link between Overconfidence and the likelihood of being a victim of online fraud, a finding that was reflected in previous studies [34–37]. Individuals may be overconfident in their ability to handle a risky situation (e.g., bullying victimization) even when the situation increases the likelihood of victimization [38]. One plausible reason is that the ability to distinguish between legitimate and fraudulent information may be lacking in overconfident people [39], and they may also be less likely than others to follow the news about financial frauds, which would reduce their awareness of the problem and leave them open to deception in the future [25, 40]. Another possibility is that excessive self-assurance fosters the appearance of control, which discourages people from adopting preventative measures such as seeking financial advice, hence making them more susceptible to financial frauds [41]. This is in fact, reflected in our survey items whereby respondents somewhat agreed that they are in control of their data and privacy, and believe that they are immune to online frauds and scams. Such an attitude is worrying as fraudsters often rely on their targeted victims’ overconfidence and apply psychological tactics to manipulate their victims [35]. This may also indicate that the respondents may somewhat lack digital privacy awareness despite claiming otherwise, or they may perceive that they are too savvy to be taken advantage of. This, therefore, warrants further investigation.

Social Influence predicts online fraud victimization, in line with previous studies that found the factor to positively affect online fraud victimization [42, 43]. In this study, Social Influence refers to one’s tendency to be influenced, manipulated and overly trusting others–a tendency that results in a higher odd of being victimized online. Similar patterns of observations were reported in other cybercrime studies such as gang membership and offending desistance [44], cyberbullying [45], and adolescent engagement in risky behaviors associated with health risk or illegal behavior [46]. This indicates the impact of an individual’s psychological nature in being highly susceptible to online frauds. Research has shown that people high in agreeableness trait are more likely to fall victim to frauds as they may be reluctant to question or challenge information presented to them [47, 48]. Similarly, those who are more susceptible to manipulation, perhaps due to a lack of knowledge or experience, may be more likely to be taken advantage of. Educational programmes and regular reminders and awareness campaigns may help in ensuring such vulnerable individuals to be more vigilant online.

In terms of demographic, being a female implies a higher tendency to be victimized online compared to males. This could be due to a combination of social, psychological, and behavioral factors. Studies suggest that women tend to engage more frequently in online activities such as social networking and e-commerce such as online shopping [49], which can expose them to a higher number of potential threats. In fact, according to Statista [50], more than 60% of women reported discovering products through social media compared to more than 50% of men in 2023. Additionally, social engineering tactics used by fraudsters [3], such as emotional manipulation or trust-building, may disproportionately affect females, who are often perceived as more empathetic or community-oriented [51]. This aligns with findings by [52] who highlight that women are more likely to engage in empathetic and trusting online behaviors, which may make them more likely to fall victim to schemes like phishing or romance scams. Furthermore, studies by Anderson [53] and Leukfeldt et al. [54] emphasize that fraudsters may exploit gender stereotypes, assuming that women are less familiar with technical aspects of online security, making them easier targets.

This study found that falling victim to online frauds was not significantly predicted by having a high level of Bank Trust. This finding is consistent with earlier research [48, 55], however it is at contrast with Farkhondeh et al. [56]. Likewise, this observation supports the notion that trust did not predict fraud vulnerability [57]. One probable explanation is that Internet fraudsters or scammers frequently used strategies such as using the names of reputable companies or people and sending fictitious messages to their targeted victims [58]. People will specifically lose trust in an institution if they think genuine messages are fraudulent ones, in addition to financial losses. The victimization of online frauds may therefore not be significantly predicted by Bank Trust.

Similarly, Self-awareness did not significantly predict online fraud victimization. This concurs with [59], however in contrast with [60, 61]. This could be attributed to the high level of digital privacy awareness among our respondents (81.9%; 83.2%). Respondents are mainly urbanite educated individuals who are also digitally active, with intermediate and advanced levels of literacy for technology, fact checking and fake news, therefore they may be more knowledgeable in terms of digital privacy and Internet safety, a pattern that was reflected in [62]. This probably explains why Self-awareness was insignificant in predicting online fraud victimization.

Safe Practice was found to be insignificant in mitigating online fraud victimization in this study, in contrast to some other studies [63–65]. The mean score (i.e., 3.41, Table 2), suggests that most respondents across both studies displayed a moderate to high level of agreement in exercising caution and adhering to good online practices. This indicates that most of the respondents are already somewhat cautious in their online behavior. Furthermore, given that a significant portion of the respondents belong to the younger generation, they are likely to be more tech-savvy and familiar with adopting standard online safety measures. Their digital fluency and regular exposure to online environments may have contributed to the widespread practice of basic security protocols, thus diminishing the predictive power of Safe Practice as a unique factor in preventing online fraud. Essentially, Safe Practice may not have emerged as a significant predictor because it is already widely implemented by this demographic, making other factors more relevant in explaining variations in fraud victimization.

## Conclusion

This study explored and determined critical predictors predicting online fraud victimization. Using the SCT as the foundational theory for the framework development, EFA was conducted, resulting in the identification of five distinct independent variables: Safe Practice, Overconfidence, Social Influence, Bank Trust, and Self-awareness. Key findings related to our two main RQs show that most of the respondents sampled are aware of digital privacy, however knowledge alone does not prevent them from becoming a fraudulent victim online. Findings show that the propensity for an individual to be influenced by their social environment (Social Influence), high level of confidence (Overconfidence) and being female significantly increase the risk of falling victim to online frauds. This highlights the need for targeted interventions such as improving critical thinking skills, enabling individuals to better analyze and evaluate the credibility of information they encounter online. Further, fostering skepticism towards online communications can empower people to recognize and resist fraudulent schemes more effectively. Finally, gender-sensitive prevention programs can be designed to address the unique ways in which different genders, especially women might experience and respond to online scams, thus ensuring more inclusive and effective protective measures.

This study has limitations that need to be acknowledged. The study used a survey methodology to gather the quantitative data needed, but this method lacks the depth or richness of data that can only be obtained through longitudinal research using qualitative data, which can produce a stronger inference of causality [66]. Further, the sample is deemed skewed as many respondents were young, educated and from urban areas. This may have affected the outcome of the study. Further, digital privacy awareness as well as literacy levels were assessed based on self-perceptions, hence this may not reflect the picture. Future studies could explore other methods to determine individuals’ awareness and their literacy levels (e.g., using experiments). Finally, the study may also lack the generalizability desired as the respondents are of Malaysian origin. Future studies involving participants from various backgrounds or nationalities may yield similar or contrasting results that are worth pursuing.

## Supporting information

S1 Appendix Survey questionnaire. (DOCX)

S1 File Inclusivity in global research. (DOCX)
