# Supplementary material for: Personal, environmental and behavioral predictors associated with online fraud victimization among adults
Source: PLoS One. 2025 Jan 29;20(1):e0317232. doi: 10.1371/journal.pone.0317232 (PMC11778706; doi:10.1371/journal.pone.0317232)
Supplement: S1 Appendix — (DOCX) [file pone.0317232.s001.docx]

# Digital Privacy and Online Fraud: Awareness, Attitude and Behaviour

The study intends to investigate the level of digital privacy awareness, attitude and behavior with regards to online fraud. Be assured that your responses will be treated with ***extreme confidentiality.*** Thank you

**Note:** Respondents to be more than 18 years’ old and Malaysians

**Part A:** Demographic

**Age: Gender:** ☐ Male ☐ Female

**Status:** ☐ Tertiary student (college/university etc.) ☐ Working ☐ Others:

**Ethnicity**: ☐ Malay ☐ Chinese ☐ Indian ☐ Others:

1. How often do you use your mobile phone in a day (hours)? (calls, apps etc.)
   - < 1 ☐ 1 – 5 ☐ More than 5
2. How often do you use social media applications in a day (hours)?
   - < 1 ☐ 1 – 5☐ More than 5
3. Are you aware of digital privacy?
   - Yes ☐ No ☐ Not sure
4. Have you been a victim of any online fraud activity (e.g., scammed, data breach, identity theft etc.)? If no, go to Q6.
   - Yes ☐ No ☐ Not sure
5. What media was used for (4) above?
   - Phone call/SMS ☐ Social media ☐ 3^rd^ party app ☐ Chat applications ☐ Others:
6. Has anyone tried to cheat/scam you before?
   - Yes ☐ No
7. Do you know anyone close who is a victim of any online fraud activity (e.g., scammed, data breach, identity theft etc.)?
   - Yes ☐ No
8. My digital literacy (i.e., ability to use technology, understanding content, handling fake news etc.) is: Familiarity with technologies (i.e., social media, messengers, apps etc.)
   - Beginner ☐ Intermediate ☐ Advanced

Familiarity with fake news (i.e., can identify fake news)

- - Beginner ☐ Intermediate ☐ Advanced Familiarity with fact-checkers (i.e., website to verify information)
  - Beginner ☐ Intermediate ☐ Advanced

1. Rate your online risky behavior level (e.g., not adopting safety measurement, disclosing personal details etc.).
   - Low ☐ Intermediate ☐ High

# Part B

Please answer all the questions below based on **YOUR personal experience and perception.**

1. Please indicate your agreement/disagreement level for the statements below using the scale provided: Strongly disagree = 1 disagree = 2 Neutral = 3 Agree =4 Strongly agree = 5

| **No** | **Statements** | 1 | 2 | 3 | 4 | 5 |
| --- | --- | --- | --- | --- | --- | --- |
| **A** | **Attitude** |  |  |  |  |  |
| 1 | I trust people easily |  |  |  |  |  |
| 2 | The Internet is safe |  |  |  |  |  |
| 3 | Online banking is safe |  |  |  |  |  |
| 4 | I trust the safety mechanisms adopted by my banks |  |  |  |  |  |
| 5 | I will never fall victim as I am always careful |  |  |  |  |  |
| 6 | I am not aware of how to setup my privacy setting |  |  |  |  |  |
| 7 | I do not care about my privacy setting |  |  |  |  |  |
| 8 | I do not mind downloading apps with APK |  |  |  |  |  |
| 9 | My family and friends influence the choice of apps that I use |  |  |  |  |  |
| 10 | I am easily manipulated and naive |  |  |  |  |  |
| 11 | I believe I can control my privacy online |  |  |  |  |  |
| 12 | I am confident that my online data will remain private and confidential. |  |  |  |  |  |
| 13 | Scammers usually target older generations only |  |  |  |  |  |
| **B** | **Behaviour** |  |  |  |  |  |
| 15 | I change my passwords regularly |  |  |  |  |  |
| 16 | I always use a strong password |  |  |  |  |  |
| 17 | I share my passwords with trusted individuals |  |  |  |  |  |
| 18 | I use auto save password settings on my phone |  |  |  |  |  |
| 19 | I always write down all my passwords in my phone notes |  |  |  |  |  |
| 20 | I use the same password for every account |  |  |  |  |  |
| 21 | I always perform 2-step verification for my personal accounts |  |  |  |  |  |
| 22 | I browse online using a secure VPN |  |  |  |  |  |
| 23 | I click on online links as they are generally safe |  |  |  |  |  |
| 24 | I do not access my banking account using public Wi-Fi |  |  |  |  |  |
| 25 | I do not perform online shopping using public Wi-Fi |  |  |  |  |  |
| 26 | I tend to disclose personal information on social media |  |  |  |  |  |
| 27 | I make sure that my chat messages online are encrypted |  |  |  |  |  |
| 28 | I use privacy settings to block malicious websites |  |  |  |  |  |
| 29 | I usually do not logout from mobile apps/social media/websites etc. |  |  |  |  |  |
| **C** | **Awareness** |  |  |  |  |  |
| 30 | I know the importance of safety measures online |  |  |  |  |  |
| 31 | I know that downloading suspicious app with APK is dangerous |  |  |  |  |  |
| 32 | I know the dangers of digital footprints |  |  |  |  |  |
| 33 | I know the dangers of sharing sensitive information online |  |  |  |  |  |

| 34 | I am aware that malicious websites may lead to identity theft (by installing a software  on my PC without my notice to collect personal info) |  |  |  |  |  |
| --- | --- | --- | --- | --- | --- | --- |
| 35 | I am aware that there are a lot of fake websites on the internet |  |  |  |  |  |
| 36 | I know which suspicious pop-up messages on websites to be ignored |  |  |  |  |  |
| 37 | I am aware of the after-effect of accepting cookies on websites |  |  |  |  |  |
| 38 | I am aware that my online activities can be tracked without my permission |  |  |  |  |  |
| 39 | I am aware that my online activities can be tracked without my knowledge |  |  |  |  |  |
| 40 | I am aware that my personal data are collected online |  |  |  |  |  |

-THE END-
